# Development of antimicrobial biomaterials produced from chitin-nanofiber sheet/silver nanoparticle composites

**DOI:** 10.1186/s12951-014-0049-1

**Published:** 2014-12-03

**Authors:** Vinh Quang Nguyen, Masayuki Ishihara, Jun Kinoda, Hidemi Hattori, Shingo Nakamura, Takeshi Ono, Yasushi Miyahira, Takemi Matsui

**Affiliations:** Faculty of System Design, Tokyo Metropolitan University, 6-6 Asahigaoka, Hino, Tokyo 191-0065 Japan; Research Institute, National Defense Medical College, 3-2 Namiki, Tokorozawa, Saitama 359-1324 Japan; Department of Oral and Maxillofacial Surgery, National Defense Medical College, 3-2 Namiki, Tokorozawa, Saitama 359-8513 Japan; Department of Global Infectious Diseases and Tropical Medicine, National Defense Medical College, 3-2 Namiki, Tokorozawa, Saitama 359-8513 Japan

**Keywords:** Antimicrobial biomaterials, Chitin nanofiber sheets, Silver nanoparticles, Wound dressings, Anti-virus sheets

## Abstract

**Background:**

Chitin nanofibers sheets (CNFSs) with nanoscale fiber-like surface structures are nontoxic and biodegradable biomaterials with large surface-to-mass ratio. CNFSs are widely applied as biomedical materials such as a functional wound dressing. This study aimed to develop antimicrobial biomaterials made up of CNFS-immobilized silver nanoparticles (CNFS/Ag NPs).

**Materials and methods:**

CNFSs were immersed in suspensions of Ag NPs (5.17 ± 1.9 nm in diameter; mean ± SD) for 30 min at room temperature to produce CNFS/Ag NPs. CNFS/Ag NPs were characterized by transmission electron microscopy (TEM) and then tested for antimicrobial activities against *Escherichia* (*E*.) *coli*, *Pseudomonas* (*P*.) *aeruginosa*, and H1N1 influenza A virus, three pathogens that represent the most widespread infectious bacteria and viruses. Ultrathin sectioning of bacterial cells also was carried out to observe the bactericidal mechanism of Ag NPs.

**Results:**

The TEM images indicated that the Ag NPs are dispersed and tightly adsorbed onto CNFSs. Although CNFSs alone have only weak antimicrobial activity, CNFS/Ag NPs showed much stronger antimicrobial properties against *E. coli*, *P. aeruginosa*, and influenza A virus, with the amount of immobilized Ag NPs onto CNFSs.

**Conclusions:**

Our results suggest that CNFS/Ag NPs interacting with those microbes exhibit stronger antimicrobial activities, and that it is possible to apply CNFS/Ag NPs as anti-virus sheets as well as anti-infectious wound dressings.

## Background

Chitin/chitosan is second most abundant natural nontoxic biomaterial, and is produced from the exoskeleton of sea food, shellfish, crabs, shrimps, insects, edible mushrooms, and sea weed algae [1]. The advantages in biochemical activities of chitin/chitosan-based materials include: anti-infectious activity [2]; stimulation of angiogenesis/wound repair; and stabilization/activation of growth factors [3–7]. Since chitin nanofiber sheets (CNFSs) are biodegradable and exhibit large surface-to-mass ratios, CNFSs are widely applied in pharmaceuticals as composite materials. The favorable properties of CNFS-based materials are enhanced as sizes of their fibers are decreased across the range of 1-100 nm [8]. In case of cosmetic dermatology, chitin nanofibrils do not only protect corneocytes and intracorneal lamellae, but also helping to maintain cutaneous homeostasis. In addition, CNFSs neutralize the activity of free radicals and trap them in their structure, thereby regulating correct cell turnover [9]. The positive charges on the surface of the fibers, along with the chelating capacity of the acetamido groups of the chitin/chitosan molecule, play important roles in adsorption of heavy metals [10,11]. Our previous study demonstrated that chitin powder with nanoscale fiber-like surface structures can adsorb Ag NPs more efficiently than chitin powder with flat/smooth film-like surface structures [12]. CNFSs have attracted much attention for application as components of pharmaceutics such as drug carriers, textile materials, sutures, and scaffold materials for tissue engineering [13,14].

Ag NPs have strong antimicrobial activity against most microorganisms, including bacteria, fungi, and viruses. In recent publications, we demonstrated that chitin/chitosan/Ag NP composites have enhanced antimicrobial activities against microbial pathogens, including bacteria (*E. coli*), fungi (*Aspergillus niger*), and virus (H1N1 influenza A virus) [15–17]. The bactericidal activity of Ag NPs is believed to result from Ag NP interactions with the cell wall, permitting Ag NPs to penetrate the membrane, thereby leading to the cell death [18]. In addition, silver ions released from Ag NP surface are thought to bind to sulfhydryl groups, leading to protein denaturation [19]. Furthermore, silver ions have been shown to penetrate through ion channels without causing damage to the cell membranes, where the ions denature the ribosome and suppress the expression of enzymes and proteins essential to ATP production [20]. Silver ions can interact with the bases in DNA causing loss of replication [21,22]. Ag NPs also induce the formation of free radicals, which in turn damage the membrane and cause cell death, formation of bactericidal reactive oxygen species (ROS), and lactate dehydrogenase activity involved in the respiratory chain [23]. Bacterial DNA can be affected by ROS, resulting in the production of superoxide anion (O_2_^-^), hydroxyl radical (OH^.^), and singlet oxygen (^1^O_2_) with subsequent oxidative damage [23,24].

Questions have been raised about the safety of prolonged use of Ag NPs in humans and animals. Generally, silver does not adversely affect mammalian cell viability. Hence, silver has been incorporated into various materials and used in antimicrobial materials to protect from infectious contamination [25]. Recently, *in vitro* studies in human cells have reported that Ag NP exposure induces metabolic arrest rather than cell death, and that human cells have a greater resistance to the toxic effects of Ag NPs in comparison with those from other organisms [26,27]. Furthermore, previous studies have revealed that while Ag NP-containing chitosan-based wound dressings are cytotoxic *in vitro*, such wound dressings perform satisfactorily *in vivo* [28]. The aim of the present work is to evaluate the bactericidal (against *E. coli* and *P. aeruginosa*) and antiviral (against influenza virus H1N1) activities of CNFS/Ag NPs, for potential biomedical applications such as wound dressings and antivirus sheets.

## Results

### Characterization of Ag NPs and CNFS/Ag NPs

**Figure 1:**
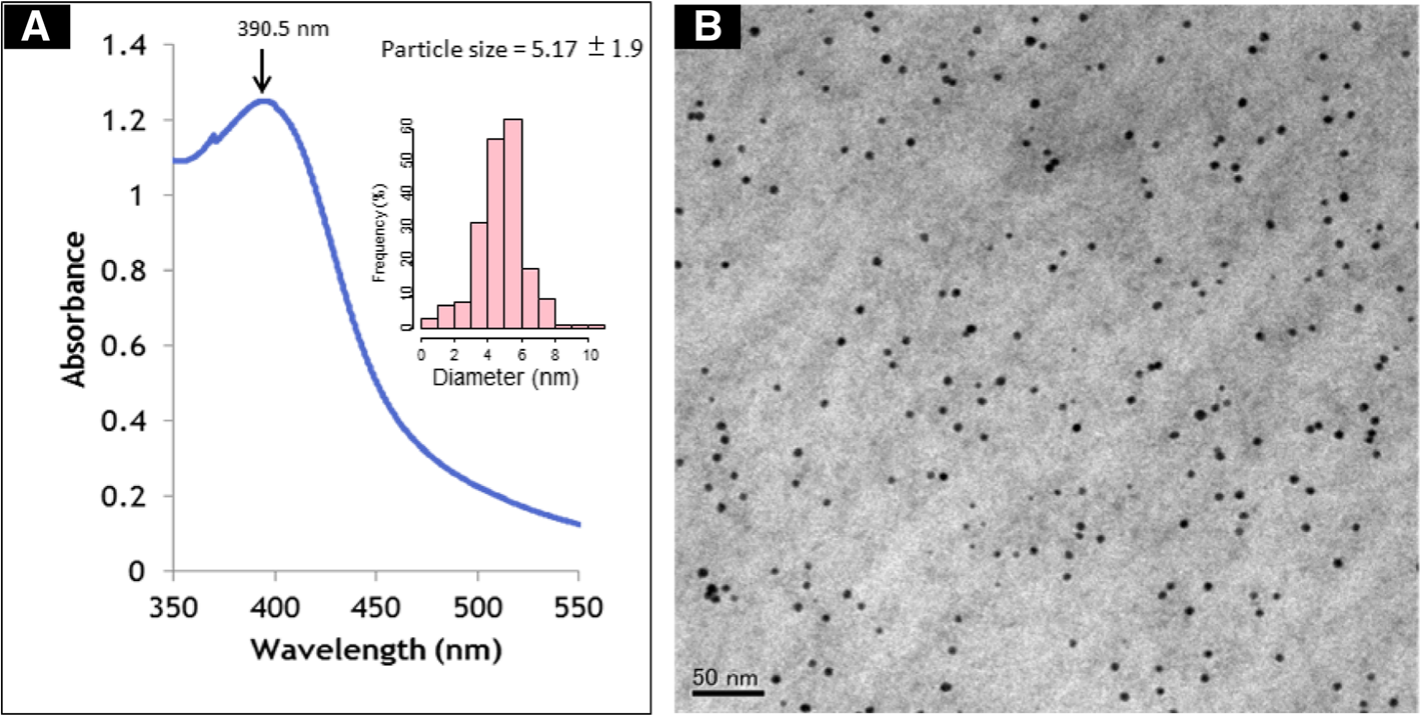
**Absorption of Ag NPs to ANSF. (A)** The UV-visible absorption spectrum of Ag NPs. The peak of absorbance at the wavelength of 390.5 nm is indicated. The inset figure is the particle size distribution histogram of the Ag NPs. **(B)** TEM micrograph of Ag NPs. Scale bar represents 50 nm.

Ag NPs were synthesized by autoclaving (at 121°C and 20 kPa) a mixture of only three components: silver-containing glass powder, glucose, and water [17,29]. TEM images showed that the Ag NPs were spherical with the average particle size of 5.17 ± 1.92 nm (mean ± SD). The results of UV-Vis analysis of the Ag NPs suspension revealed that the peak at 390.5 nm is representative of the Ag NPs in this study (Figure 1).

**Figure 2:**
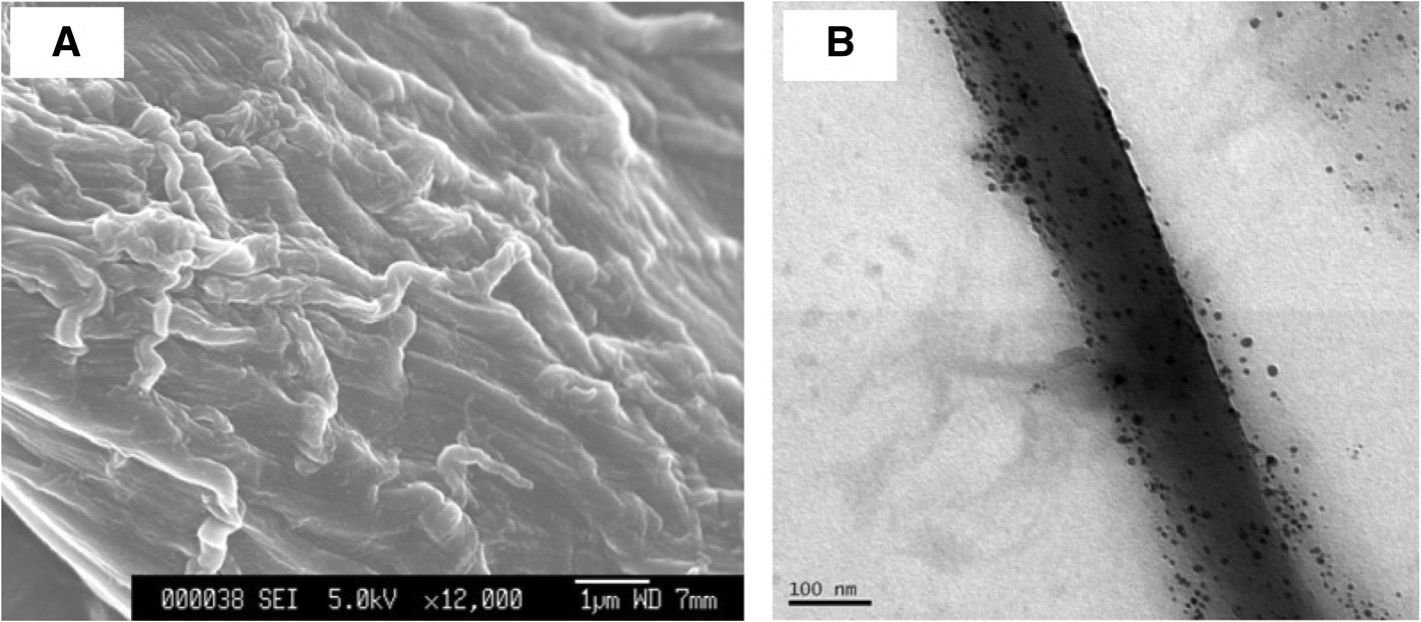
**SEM and TEM images of CNFS.** SEM image of CNFS; scale bar represents 1 μm **(A)**. TEM image of CNFS/Ag NPs composite sheet; scale bar represents 100 nm **(B)**.

**Figure 3:**
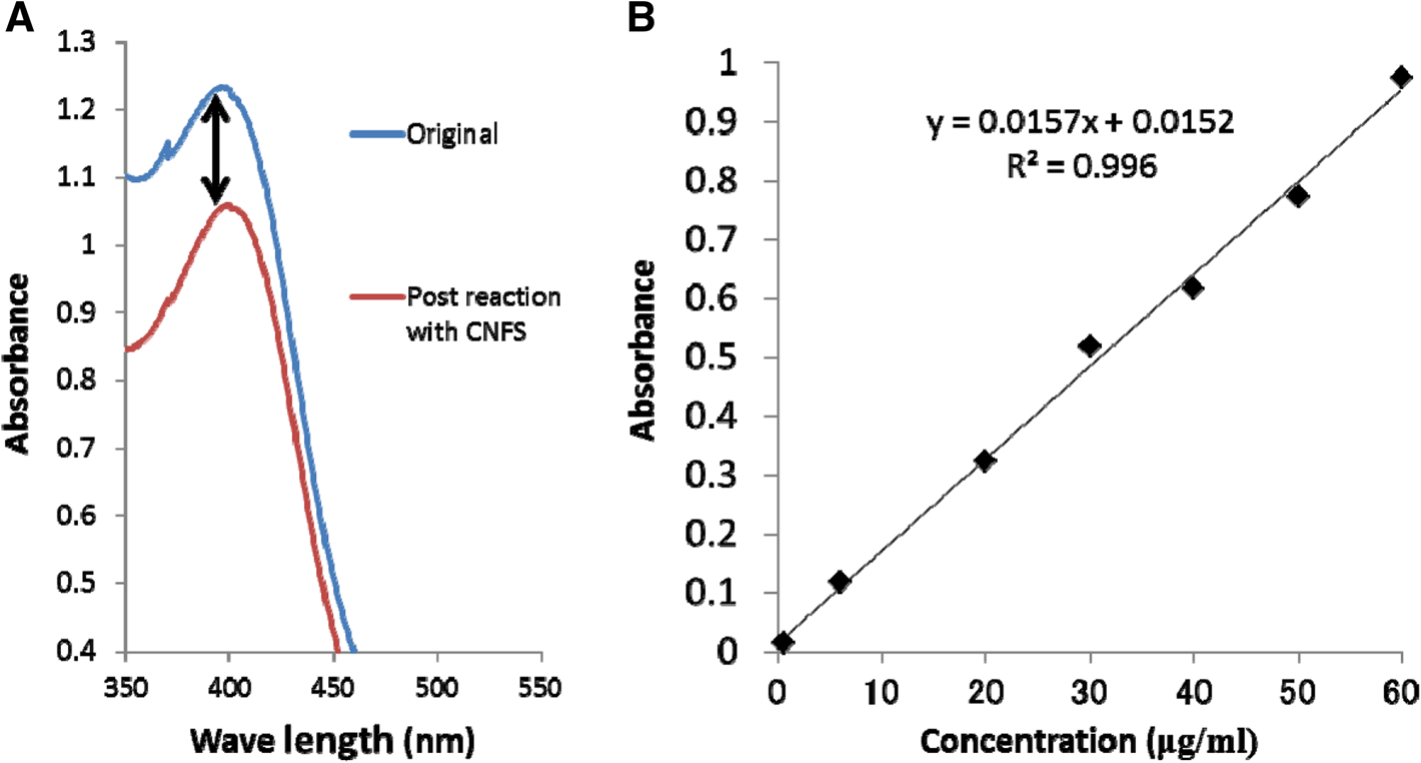
**The absorbance spectra of Ag NPs.** The absorbance spectra of original Ag NP suspension (blue line) and suspension after reaction with 1 cm^2^ CNFS (red line) **(A)**. The relationship between absorbance at 390.5 nm and the concentration of Ag NPs in the suspension **(B)**.

The surface morphology of the CNFS has been characterized using SEM imaging. The CNFS has a nanoscale fiber-like surface structure (Figure 2A). TEM observation of CNFS/Ag NPs revealed that the Ag NPs were stably adsorbed to the surface of CNFS (Figure 2B). Based on comparison of absorbance values of Ag NP suspension before and after reaction with CNFS, along with the equation for the standard curve of absorbance at 390.5 nm as a function of the concentration of Ag NPs in suspension, we estimated that Ag NPs were immobilized on CNFS at 8. 45 μg per cm^2^ (Figure 3A and Figure 3B).

### Bactericidal activity of CNFS/Ag NPs

**Figure 4:**
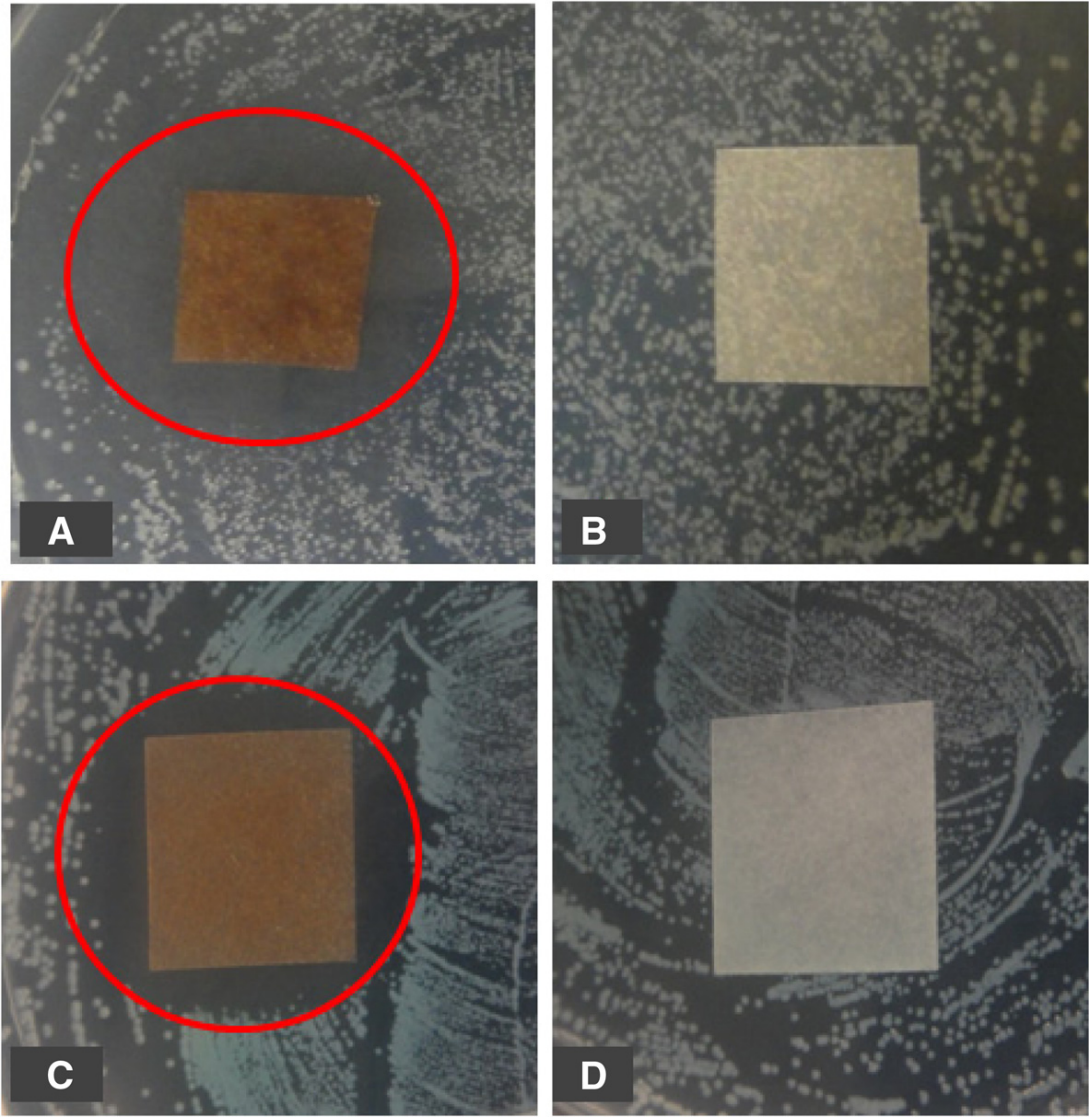
**Antimicrobial activity of CNFS/Ag NPs against** ***E*** **.** ***coli*** **(A); CNFS alone against** ***E*** **.** ***coli*** **(B); CNFS/Ag NPs against** ***P*** **.** ***aeruginosa*** **(C); and CNFS alone against** ***P*** **.** ***aeruginosa*** **(D).** The CNFS/Ag NPs showed inhibition zone of bacterial growth as shown with red circles (A and C), although the CNFS alone exhibited no detectable inhibitory activity against either bacterial species (B and D).

To establish the bactericidal properties of CNFS/Ag NPs, the sheet were tested with two infectious bacteria: *E. coli* and *P. aeruginosa*. The inhibition zone of bacterial growth around CNFS/Ag NPs and CNFS alone against *E. coli* and *P. aeruginosa* are shown in Figure 4. There was no zone of growth inhibition around CNFS alone for either *E. coli* or *P. aeruginosa*. With CNFS/Ag NPs (8.5 μg/ml), there were clear zones of inhibition of ≈ 30 mm diameter (for *E. coli*) and ≈ 25 mm diameter (for *P. aeruginosa*) around CNFS/Ag NPs after 24 h incubation (Figure 4).

**Figure 5:**
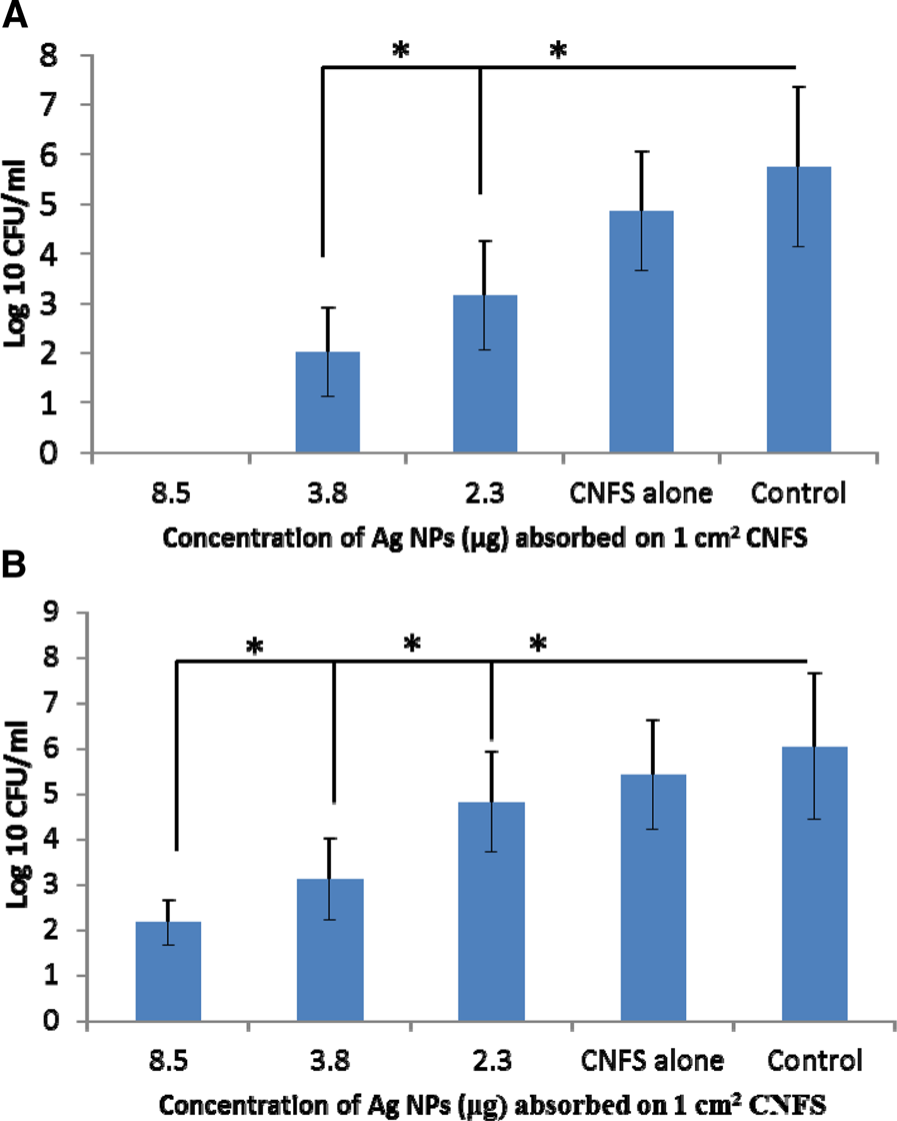
**Bactericidal activities of CNFS**/**Ag NPs against** ***E. coli*** **(A),** **and** ***P. aeruginosa*** **(B) at different concentration of Ag NPs.** Data are mean value ± standard deviation (n = 6); the asterisk (*) indicates a statistically significant difference (p <0.01) using two-sample t-test.

Bactericidal tests of CNFS/Ag NPs were performed against *E. coli* and *P. aeruginosa* by counting the viable bacterial colonies after treatment with different concentrations of Ag NPs immobilized on CNFS (2.3, 3.8, 8.5 μg/1 cm^2^ CNFS). Samples of *E. coli* were completely eradicated when exposed to CNFS contained 8.5 μg/ml of Ag NPs. The high concentration Ag NPs immobilized on CNFS gave significant decreases of cell number in log 10 CFU/ml, while CNFS alone gave only a little decrease against *E. coli* and *P. aeruginosa*. Thus, the bactericidal activity of CNFS/Ag NPs increased with increased Ag NP loading (Figure 5).

### Antiviral activity of CNFS/Ag NPs

**Figure 6:**
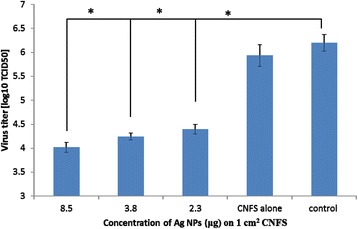
**Antiviral activity of CNFS/** **Ag NPs against H1N1** ***influenza A virus.*** The viruses after treated with CNFS/Ag NPs were grown and their titers were determined with MDCK cells. At concentration of Ag NPs of 8.5 μg/1 cm^2^ CNFS, there was a reduction of greater than 2 log10 (100-fold) corresponding to reduction of viral titers by an approximate 99%. Data are mean value ± standard deviation; n = 3; the asterisk (*) represents statistically significant difference (p <0.01) using two-sample t-test.

In order to confirm the antiviral activity of CNFS/Ag NPs, the CNFSs carrying various amounts of immobilized Ag NPs were evaluated for antiviral activity for human influenza A virus (A/PR/8/34 (H1N1)). The high concentration Ag NPs immobilized on CNFS gave significant decreases of virus number in log 10 CFU/ml, while CNFS alone gave only a little decrease against influenza A virus. At concentration of Ag NPs of 8.5 μg/1 cm^2^ chitin sheet, there was a reduction of greater than 2 log10 (100-fold) corresponding to reduction of viral titers by approximate 99% (Figure 6). This mentions that the antiviral activities of the CNFS/Ag NPs sheet were due to the interaction between virions and Ag NPs. The viruses may be adsorbed and immobilized on the CNFS/Ag NPs sheet. Therefore, inceasing amount of nAg on the chitin sheet makes more viruses adsored and immobilized on the CNFS/Ag NPs sheet.

### Ultrathin sectioning of bacterial cells

**Figure 7:**
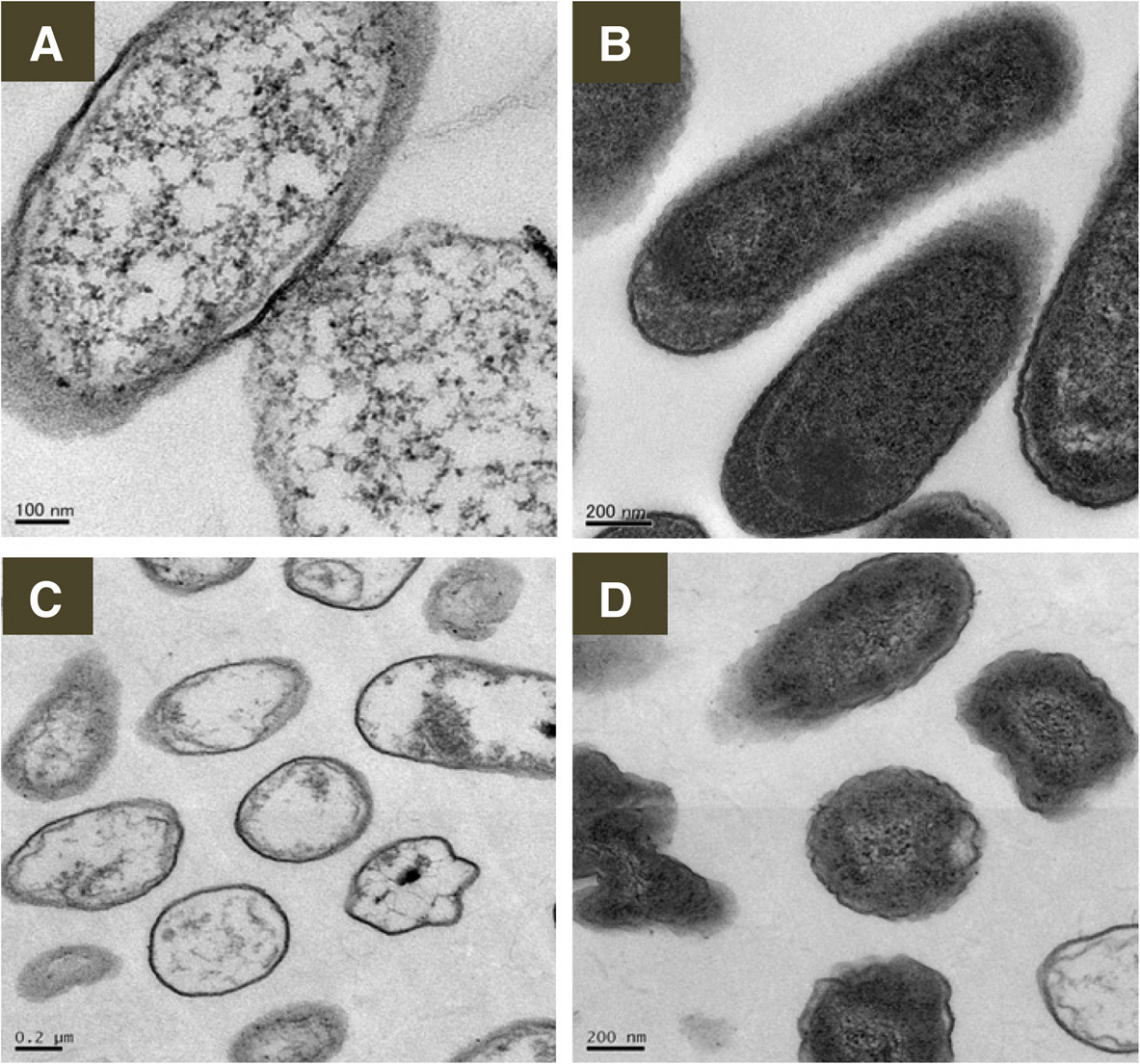
**Representative TEM images of morphology and structure of** ***E. coli*** **and** ***P. aeruginosa*** **. (A)** *E. coli* treated with Ag NPs; **(B)** normal *E. coli*; **(C)** *P. aeruginosa* treated with Ag NPs; **(D)** normal *P. aeruginosa*. Exposure to Ag NPs resulted in damage to the structure of bacterial cell membranes, condensed DNA, and coagulated cytoplasmic components. Normal bacterial cells were smooth, exhibiting intact surfaces and undamaged structures of inner membranes. Scale bars are as indicated.

The mechanism(s) of bactericidal activity of Ag NPs remain poorly understood. Ultrathin sectioning was carried out to obtain further understanding of the bactericidality and the interaction of the Ag NPs with bacterial cells. After 1 h treatment with a suspension of Ag NPs, the cytoplasmic components of *E. coli* and *P. aeruginosa* were coagulated, leading to vacant spaces within the cells. Gross inspection of the TEM images revealed non-homogeneity of the cytoplasm in the Ag NP-treated cells compared with the controls (Figure 7). The density of cytoplasmic components in treated cells was obviously decreased compared with the control. Plasma membranes of treated cells were detached from the cell wall, leaving open spaces between the membrane and cell wall. Furthermore, DNA was condensed (Figure 7).

## Discussion

Several kinds of materials are used for wound dressings, including cotton, chitin, chitosan, alloskin, pigskin, and other biologic-based materials. The various materials are commonly used in clinical settings, but these dressings often have some disadvantages such as low antimicrobial activity, allergenicity, toxic effects, and poor adhesiveness [8–10]. In the present study, we developed a potential wound dressing composed of Ag NPs (5.17 ± 1.9 nm in diameter) immobilized on CNFS to remedy some of the disadvantages of current wound dressings. CNFS was combined with Ag NPs, which act as a barrier to microorganisms, thereby limiting cross contamination. The Ag NPs were homogeneously dispersed and tightly immobilized on CNFS. The CNFS/Ag NPs showed strong antimicrobial activity against *E. coli*, *P. aeruginosa*, and influenza A virus.

The Ag NPs used in this research were produced using environmental-friendly materials and processes to control the size of Ag NPs, yielding Ag NPs of ≈ 5 nm in diameter. Components of the NPs included silver-containing glass powder, glucose, and water. Silver-containing glass powder often is used in osteal or dental applications as an antimicrobial agent; glucose has the advantage of being an environmentally friendly agent [29]. In previous work, we have demonstrated that the antimicrobial activity of Ag NPs depended on particle size [12,15,17]. Ag NPs of small size (3-10 nm diameter) have strong antimicrobial activity against *E. coli*, *P. aeruginosa*, and influenza A virus, and it is hypothesized that Ag NPs with smaller particle sizes have larger available surface areas for interaction with microorganisms [17,29].

The CNFS used in this study has a nanoscale fiber-like surface structure, with corresponding increases in the available surface area for adsorption of Ag NPs. In addition, the advantages in biochemical activities of chitin/chitosan-based materials include anti-infectious activity [2], stimulation of angiogenesis/wound repair, and stabilization/activation of growth factors [3–7]. Recent studies show that the application of CNFS to skin improved the epithelial granular layer and increased granular density, suggesting the potential use of CNFS as a component of skin-protective formulations [30]. CNFS also has been shown to inhibit mucosal inflammation by suppressing the MPO-positive cells such as leukocytes [31].

This study used CNFS, a commercially available wound dressing, in combination with Ag NPs to provide stronger antimicrobial ability. The composite thus might serve as a new biocompatible wound dressing with reduced danger of cross contamination. CNFS/Ag NPs showed much stronger bactericidal activity against *E. coli* and *P. aeruginosa*, with clear zones of inhibition around the sheet. We also observed antiviral activity against influenza A virus, presumably due to the interaction between virions and Ag NPs immobilized on CNFS. Therefore, increasing amounts of Ag NPs on CNFS may further increase the number of virions immobilized on CNFS/Ag NPs, yielding increased virucidal activity. Our results suggest that CNFS/Ag NPs can be applied not only as functional wound dressings, but also as antimicrobial agents, including antivirus sheets.

CNFSs containing 8.5 μg Ag NPs/1 cm^2^ sheet (7.3 ± 0.1 mg) completely eradicated *E. coli*. Several potential mechanisms have been reported for the bactericidal activities of Ag NPs [21,22]. Those studies showed that Ag NP exposure resulted in decreased density of cytoplasmic components, condensation of bacterial DNA, and disorganization of the cytoplasmic membrane with detachment of the plasma membrane from the cell wall. These phenomena suggested that Ag NPs induced a loss of integrity of the cytoplasm and membranes, causing malfunction of organelles and membranes, and leading to cell death. Alternatively, bacterial DNA could be affected by ROS, resulting in the production of superoxide anions (O_2_^-^) with subsequent oxidative damage [23]. This study is required to carry out biochemical analyses to confirm those mechanism.

The effect of the size of Ag NPs on antiviral activity suggests that various viruses interact selectively with smaller (≤10 nm diameter) Ag NPs, as previously reported for HIV-1 [32] and hepatitis B viruses [33]. We also previously reported a size-dependence for the effect of free Ag NPs on antiviral activity against influenza A virus [29]. In the context of anti-influenza A virus activity, further spatial restriction due to the CNFS would be expected to prevent or weaken the interaction between virions and Ag NPs. Although the virus was not completely eradicated when exposed to CNFS/Ag NPs, the adsorption of Ag NPs onto CNFS provided stronger antivirus activity. Thus, the interaction between the virions and the Ag NPs is expected to be further increased with increasing amounts of Ag NPs on the CNFSs.

## Conclusions

Our TEM image analysis indicated that Ag NPs are dispersed and tightly adsorbed on CNFS. Our antimicrobial assays further demonstrated that Ag NPs immobilized on CNFS provide much higher antimicrobial activities against *E. coli*, *P. aeruginosa*, and influenza A virus. Thus, we propose that CNFS/Ag NPs might find use as anti-influenza sheets as well as anti-bacterial wound dressing sheets.

## Materials and methods

### Materials

Silver-containing glass powder (BSP21, Ag content: 1 wt%; average grain size: 10 μm) was purchased from Kankyo Science (Kyoto, Japan). CNFSs (degree of deacetylation: ≈ 30%) used in this study were obtained as the commercial product (BeschitinW, Unichika Ltd., Tokyo, Japan). d-Glucose was purchased from Wako Pure Chemical Industries, Ltd. (Osaka, Japan). All chemicals were used as received.

### Preparation of Ag NPs

A suspension of size-controlled Ag NPs was prepared as previously described [29]. Briefly, 0.5 g of Ag-containing glass powder was dispersed in 50 ml of an aqueous solution of 0.8 wt% glucose in a 100 ml glass vial. The mixture was autoclaved at 121°C and 200 kPa for 20 min and then gradually cooled to room temperature; the mixture then was centrifuged at 1500 g for 10 min. The resulting brown supernatant containing the Ag NP suspension was stored in the dark at 4°C. Transmission electron microscopy (TEM) specimens were prepared by casting a small drop of a suspension of Ag NPs onto a carbon-coated copper grid; excess solution was then removed using filter paper and the specimens were dried at room temperature. TEM images were obtained using a JEOL JEM-1010 microscope (Nihon Electronics Inc., Tokyo, Japan) operated at 80 kV. The diameter size of Ag NPs from TEM image was determined using ImageJ 1.45 software (http://rsb.info.nih.gov/ij).

### Preparation of CNFS/Ag NPs

CNFS (1 cm × 1 cm) was submerged in a 1.5 ml ClickFit polypropylene microcentrifuge tube (TreffLab AG, Degersheim, Switzerland) containing 1 ml of Ag NP suspension (at about 30 μg/ml) and shaken well for 30 min using a shaker (Mild Mixer PR-36; TAITEC, Tokyo, Japan). The post-reaction supernatant was analyzed using a UV-visible spectrometer (Jasco V-630, Tokyo, Japan) to measure the amount of unreacted Ag NPs as a peak of absorbance at wavelength of 390.5 nm. The CNFS/Ag NP composites were washed twice with distilled water. The washed composites were air dried up on a clean bench for 1 h and used in bactericidal assays on the same day. TEM inspection confirmed that the Ag NPs were homogeneously dispersed and immobilized on the CNFS, which had become brown in color. The concentrations of Ag NPs immobilized on the CNFS were calculated based on the UV-Vis spectra of Ag NPs before and after mixing with CNFS, using a standard curve of Ag NPs generated for a previous publication [15].

Scanning electron microscopy (SEM) specimens of the CNFS/Ag NP composites were mounted on metal mounts with double-sided adhesive tape and coated with gold plasma to enhance conductivity using a plasma multi-coater PMC-5000 (Meiwafosis Co., Ltd., Tokyo, Japan). The surface morphology of coated samples was examined by JSM-6340 F microscope (JEOL, Tokyo, Japan) operated at 5 kV. The TEM image of CNFS/Ag NPs were carried out by cutting the composite sheet into very small pieces and then resuspending in 200 μl distilled water; 5 μl of the resulting suspension was observed by TEM with the JEOL JEM-1010 microscope.

### Bactericidal activity of CNFS/Ag NPs

A culture of *E. coli* strain DH5α (Takara Co., Kyoto, Japan) was stored at -80°C in Luria-Bertani (LB) broth containing 50% sterile glycerol. Overnight cultures were prepared by growing a single *E. coli* colony overnight at 37°C in 5 ml of LB medium. On the next day, 200 μl of the overnight culture was inoculated into 2 ml of LB medium and incubated at 37°C for 6 h or until the optical density at 600 nm (OD_600_) reached 0.260. The *E. coli* culture then was diluted 4-fold with LB broth, and 30 μl of the diluted suspension were spread on LB agar (ForMedium Ltd., Hunstanton, England). CNFS/Ag NPs and CNFS alone then were placed onto the surface of the inoculated agar plate, which was incubated at 37°C overnight. Growth inhibition zones around the sheets were measured on the subsequent day using a centimeter scale.

The experiment on *P. aeruginosa* was carried out using essentially the same technique as for *E. coli*. The *P. aeruginosa* strain ATCC 27853 (American Type Culture Collection (ATCC), Manassas, USA) was stored at -80°C in Luria-Bertani (LB) broth containing 50% sterile glycerol. The cell suspension of *P. aeruginosa* was prepared as follows: 20 μl of stock suspension was plated onto Pseudomonas Isolation agar (Neogen Ltd. Michigan, USA) and incubated at 37°C for 18 h. Colonies then were resuspended by placing 2 ml of LB broth on the plate surface and gently shaking the plate by hand for few minutes. The resulting suspension was pipetted to a new tube for the next experiments (or stocked at -80°C with glycerol). Following adjustment to OD_600_ of 0.26, this suspension was diluted a further 4-fold, and 30 μl was spread onto nutrient agar (Nissui Pharmaceutical CO., LTD, Tokyo, Japan). CNFS/Ag NPs and CNFS alone then were placed onto the surface of the inoculated agar plate, which was incubated at 37°C overnight. Growth inhibition zones around the sheets were measured on the subsequent day using a centimeter scale.

Forty μl of the diluted cultures of *E. coli* and *P. aeruginosa* were dropped onto each CNFS composite harboring immobilized Ag NPs at various concentrations (8.5, 3.8, 2.3, and 0 μg/1 cm^2^ sheet (7.3 ± 0.1 mg)). All of the sheets were incubated at 37°C for 1 h, and each sheet then was immersed/washed in 1 ml LB medium. The resulting wash suspensions were subjected to 10-fold serial dilutions, and 50 μl samples of diluted suspensions were plated (to 90 × 15 mm petri plates of LB agar (for *E. coli*) or nutrient agar (for *P. aeruginosa*)). Plates were incubated at 37°C for 24 h, and viable cells were enumerated.

### Evaluation of the antiviral activity of CNFS /Ag NPs

Antiviral activity of CNFS/Ag NPs was evaluated against H1N1 influenza A virus as described previously [17,30]. Fifty μl of viral suspension (about 10^5^ TCID_50_/ml) was added onto CNFS/Ag NPs consisting of various amounts of Ag NPs (8.5, 3.8, 2.3, and 0 μg) immobilized on 1 cm^2^ CNFS (7.3 ± 0.1 mg). The virus-inoculated composites were placed in an empty petri dish and incubated at room temperature for 1 h to facilitate the interaction between the viruses and the CNFS/Ag NPs. The sheets then were individually transferred to 1.5 ml tubes, each of which received 450 μl phosphate-buffered saline (PBS) and 1 min of vortexing. Following centrifugation at 6400 g for 5 min, the supernatants were transferred to new tubes, then subjected to eleven 2-fold serial dilutions in PBS. Fifty μl of each diluted supernatant was added to the individual wells of a 96-well plate containing MDCK cells. The samples were incubated at 37°C and 5% CO_2_ for 1 h to allow virus adsorption to the cells. Aliquots of growth medium (50 μl DMEM medium containing 0.4% BSA and 5 μg/ml trypsin) were added to each well. After 5 days of incubation, another 50 μl of DMEM medium containing 0.4% BSA was added to each well. Seven days post-infection, surviving cells were fixed with methanol (200 μl/well, two times), and stained with 50 μl of 5% Giemsa stain solution. Cells counts (stained (uninfected) and unstained (infected)) were determined, and viral titers (in TCID_50_/ml) were calculated according to method of Reed and Muench [17,30].

### Ultrathin sectioning of bacterial cells

In order to understand the bactericidal activities of silver nanoparticles, ultrathin sectioning was carried out to observe ultrastructural changes in bacterial cells. Two ml of Ag NPs suspension (6 μg/ml) was placed on the surfaces of agar plates containing colonies of *E .coli* or *P. aeruginosa*. After 1 h the colonies were recovered and fixed overnight (minimum of 2 h) at 4°C with 2% glutaraldehyde and 2% paraformaldehyde in 0.1 M phosphate buffer, pH 7.4. The fixed samples were washed overnight (minimum of 2 h) at 4°C in 0.1 M phosphate buffer, then post-fixed for 2 h at 4°C in 1% OsO_4_ in 0.1 M phosphate buffer. The samples then were dehydrated by using a series of alcohol solutions at increasing concentration (50, 75, 95% at 20 min each, followed by 2 passages in 100% ethanol for 30 min each). Samples were infiltrated at room temperature by immersion in propylene oxide (2 × 30 min), 1 : 1 mixture of propylene oxide and epoxy resin (1 h), 1 : 2 mixture of propylene oxide and epoxy resin (overnight), and epoxy resin only (minimum 4 h). The samples then were embedded with epoxy resin in a Beem capsule and polymerized in an oven at 37°C/12 h, 45°C/24 h, and 60°C/48 h. The polymerized samples were first semi-thin sectioned at 1.5 μm with glass knives using UltraCut S and stained with Toluidine Blue. Ultrathin sections were obtained with an ultramicrotome (UltraCut S, Reichert) with ultrathin slices 60 to 90 nm in thickness. Ultrathin slices were recovered on a 3.0 mm-diameter 200-mesh copper grid and stained with uranyl acetate for 20 min and lead acetate for 1 min. The grids were examined by TEM (JEM-1010).

### Statistical analyses

Statistical analyses were carried out using StatMate III, Macintosh Version (ATMS Co., Tokyo, Japan). Statistical significance was assumed when p <0.01. Where relevant, values are provided as mean ± SD.

## Abbreviations

CNFSs: Chitin nanofibers sheets
CNFS/Ag NPs: CNFS-immobilized silver nanoparticles
TEM: Transmission electron microscopy
SEM: Scanning electron microscopy
UV-Vis: Visible-Ultraviolet spectrums
